# Supplementary material for: Antibiotic Resistance in Bacteria—A Review
Source: Antibiotics (Basel). 2022 Aug 9;11(8):1079. doi: 10.3390/antibiotics11081079 (PMC9404765; doi:10.3390/antibiotics11081079)
Supplement: Supplementary file 1 [file antibiotics-11-01079-s001.zip › antibiotics-1824894-supplementary.pdf]

Table S1. The first or latest cases of antibiotic resistance of chosen foodborne pathogens in different parts of the world

Kind of pathogens	Country	Source of isolation	Antibiotic resistance of strains	Genes responsible for resistance	References
Campylobacter spp.	Denmark	Humans, poultry, pigs	fluoroquinolones<br>macrolides<br><br>tetracyclines<br>β-lactams	*gyrA*<br>*ermB, 23S rRNA, rplD, rplV*<br>*cmeA, cmeB, cmeC cmeR*<br>*tet(O)*<br>*bla*$_{OXA-61}$	Aarestrup and Engberg [257]
	Denmark, United States	Humans	fluoroquinolones<br>macrolides	*gyrA*<br>*ermB, 23S rRNA, rplD, rplV*<br>*cmeA, cmeB, cmeC cmeR*	Engberg et al., [258]
	United Kingdom	Humans, poultry	fluoroquinolones	*gyrA*	Piddock, [259]
	Belgium, Canada, USA	Humans, sheep	tetracyclines	*tet(O)*	Taylor et al., [260]
	United Kingdom	Pigs	macrolides	*ermB, 23S rRNA, rplD, rplV*	Burridge et al., [261]
	Finland, Netherlands	Human, poultry	fluoroquinolones	*gyrA*	Rautelin et al.,[262]; Endtz et al., [263]
	United States	Human	fluoroquinolones<br>macrolides	*gyrA*<br>*ermB, 23S rRNA, rplD, rplV*	Nachamkin et. al., [264]
E.coli	Poland	Poultry litter, cloaca and meat	ampicillin, moxycyline /clavulanic acid,<br>doxycycline,<br>ciprofloxacin,<br>trimethoprim/sulfamethoxazole,<br>gentamycin	*blaTEM, blaCTX-M*<br><br>*tet(A), tet(B)*<br>*qnrA, qnrB, qnrS,*<br>*sul1, sul2, sul3, dfr1, dfr5, dfr7/17*<br>*strA-strB, aphA1, aac(3)-II*	Racewicz et al. [265]
	Nepal	Humans,	ampicillin, ciprofloxacin, nalidixic acid, norfloxacin, ceftriaxone, cefixime, imipenem and meropenem	*blaOXA-48*	Gurung et al. [266]

	Portugal, Spain, Ireland, Cyprus, Germany, Finland, Norway	Environemnt, wastewater, surface water	aminoglycosides, sulfonamides, tetracyclines, β-lactams, quinolones, amphenicols, vancomycin, multidrug resistance-MDR,	*aadA and strB, blaGES, blaOXA, and blaVEB, ereA, ermF, matA/mel, sul1, tetM and tetQ, qacEdelta1, qacH*	Pärnänen et al. [267]
	Italy	Humans	aminoglycosides, fluoroquinolones, β-lactams, including carbapenems	*blaTEM-1, blaOXA-1, blaOXA-9, blaCTX-M-15, tet(B), tet(C), armA, aac(6′)-Ib-cr, aac(3)-II, catB3, and arr3, , aac(6′)-Ib-cr, aac(3)-II, blaOXA-1, catB3, arr3 blaNDM-1*	D'Andrea et al. [268]
	Australia	Dogs	tetracycline, enrofloxacin, ciprofloxacin, streptomycin, trimethoprim/sulfamethoxazole, ampicillin, ticarcillin, piperacillin, amoxicillin/clavulanic acid, ticarcillin/clavulanic acid	*blaTEM, blaCMY-7, catA1, dfrA17-aadA5,*	Sidjabat et al. [269]
	South Africa	Poultry	doxycycline, trimethoprim-sulfamethoxazole, ampicillin, enrofloxacin	*mcr-1, mcr-2*	Hassan et al. [270]
	South Korea, China	Foods	trimethoprim-sulfamethoxazole, oxytetracycline, ampicillin, enrofloxacin, ciprofloxacin, chloramphenicol	*cmlA, cat-1, cat-2, cat-3, floR.*	Li et al. [271]
E. faecium, E. faecalis	Poland, Nederlands	Broiler chicks	lincomycin, erythromycin, tetracycline, ciprofloxacin,	*tet(M), tet(L), lnu(B), erm(B), Int-Tn, ant(4′)-Ia, ant(6′)-Ia, cat,*	Stępień-Pyśniak et al. [67]
	Italy	Swine	florfenicol, chloramphenicol, tetracycline, streptomycine, kanamycin,	*optrA, cfrD, poxtA*	Fioriti et al. [271]

				aadA1,	
	China	Cattle- dairy cows	erythromycin, tetracycline, quinupristin/dalfopristin, rifampicin, chloramphenicol, ciprofloxacin	*tet(K), tet(L), tet(M), tet(O), tet(S)* *ermA, ermB, and ermC*	Yang et al. [272]
	Saudi Arabia	Humans	lincosamide (clindamycin), macrolides (erythromycin), quinolone, tetracycline, streptogramin	*vanA, tet(M), aph(3′)-III, aac(6′)-aph(2″),aad(6) mphD, erm(B), efrA, emeA, efrB, dfrE, dfrG*	Farman et al. [273]
	Iran		tetracycline, erythromycin,	*aac(6′)-Ie-aph(2′′)-Ia, aph(3′)-IIIa, ant(4′)-Ia*	Mirnejad et al.[274]
	Southern, West and Northen Africa	Humans,	erythromycin, kanamycin, gentamycin, tetracycline, streptomycine, trimethoprim/sulfamethoxazole vancomycin,	*erm(B),tet(M), vanA*	Osei Sekyere & Mensah, [275]
		Animals	clindamycin, ciprofloxacin, penicillin, trimethoprim/sulfamethoxazole		
		Environment	clindamycin, ciprofloxacin, erythromycin, penicillin, trimethoprim/sulfamethoxazole, vancomycin,		

E. faecalis, E. faecium, E. durans	Hungary	Cheese- sheep, goat	rifampicin vancomycin teicoplanin erythromycin, minocycline, nitrofurantoin, ciprofloxacin, levofloxacin	*ermA, ermB, ermC, and msrC*	Výrostková et al. [276]
E. faecalis, E. faecium, E. hirae	North America	Beef meat	lincomycin, quinupristin-dalfopristin, tetracycline, erythromycin, ciprofloxacin	*erm(B), msrC, tet(B), tet(C), tet(L), tet(M) vanA, vanB, vanC1,*	Holman et al.[277]
E. faecalis, E. faecium, E. casseliflavus, E. hirae	South Africa-Egypt	Sheep, goats	oxacillin, vancomycin, linezolid	*blaZ, vanA, ermB, tet(M), optrA*	El-Zamkan et al. [278]
E. hirae, E. villorum, E. durans, E. faecium, E. malodoratus, E. faecalis, E. casselifavus, E. mundtii, E. avium, E. columbae, E. gallinarum	Canada	Humans, beef cattle, abattoirs, environment (streams, wastewater)	β-lactams, quinolones, tetracycline, macrolides, teicoplanin, vancomycin	*dfrE, lsa(A), , erm(B), ant(6)-Ia, aph(3')-IIIa, sat4, tet(L), tet(M), vanY, vanZ, vanA*	Zaheer et al. [279]
L. monocytogenes	France, Switzerland	Human-*meningoencephalitis*	chloramphenicol, erythromycin, streptomycin, tetracycline, minocycline	*cat221,ermAM,aad6,tet(S); tet(M)/int-Tn, tet(K), tet(L), tet(S),*	Charpentier &Courvalin,[168]
	Greece	Humans	chloramphenicol, clindamycin, gentamycin, streptomycin, tobramycin		

		Milk products	tetracycline	tet(M)	Filiousis et al. [280]
	South Africa	Environmemt-water	penicillin, cephalothin, ciprofloxacin, levofloxacin, tetracycline, trimethoprim/sulphamethoxazole, nalidixic acid, chloramphenicol, erythromycin, vancomycin, clindamycin, and oxacillin	*sulI, tet(A), catII, blaTEM, blaSHV, blaOXA-1*	Mpondo et al. [281]
	Brazil	Food, food industry	cefoxitin, nalidix acid, clindamycin	*tet(M), ermB*	Haubert et al.[183]
	United States	Natural environment, foods	cephalosporins, Streptomycin, tetracycline	*Tet(M)*	Bae et al. [186]
L. monocytogenes L. seeligeri, L. welshimeri, L. ivanovii, L. innocua		Food- catfish fillets	cefotaxime, clindamycin	*tet(M)*	Chen et al.[282]
L. monocytogenes L. innocua	Spain	ready-to-eat meat and dairy products	clindamycin, ciprofloxacin, ampicillin, tetracycline,	*mef A, msr A, erm A, erm B, lnu A, lnu B tet (M), tet(L),*	Escolar et al.[283]
L. innocua,	Italy	Food	tetracycline	*tet (K), tet(M), tet (L)*	Roberts et al. [284]
L. monocytogenes		Food, human	tetracycline, erythromycine	*tet (S)*	

L. welshimeri		Food, environment, human disease	tetracycline	*tet (S)*, erm *A*, *ermB*, *ermC*	
Salmonella spp	Uganda	Meat and milk samples	cefazolin, kanamycin, polymixin B, nitrofurantoin	*blaTem-2, aadA, bla-CTX-M-gr2, strA,*	Onohuean et al.[285]
	South Africa	Broiler chickens	tetracycline, trimethoprim/sulfamthoxazole, trimethoprim, kanamycin, gentamicin, ampicillin, amoxicillin, chloramphenicol, erythromycin, streptomycin	*tet (A), ant (3")-la, tet B*	Zishiri et al.[286]
	Brazil	Fishes	amoxicillin/clavulanic acid, tetracycline, sulfonamide, chloramphenicol	*Tet(A), floR, sul2,*	Fereira et al.[287]
	Poland	Pork, poultry	penicillins, cephalosporins, aminoglycosides, fluorochinolones, sulfonamides, tetracyclines	bla_{CMY-2}, bla_{PSE-1} *a*, bla_{TEM}, bla_{PSE-1}, *sul2, adfR, floR, tet(A), tet(B),*	Pławińska-Czarnak et al.[288]
	United States	Chicken, swine, turkey,	amoxicillin-clavulanic acid, ampicillin, cephalothin, cefoxitin, ceftiofur, ceftriaxone	*blaCMY, blaTEM, aphAI-IAB, strA, aadA1, aadA2, sul1, sul2, and/or sul3,*	Lyne et al.[289]
S. Typhimurium, S. Typhi, S. Enteritidis, S. Rissen, S. Derby	Italy	Pigs, slaughterhouse, pork meat	aminoglycoside (amikacin and tobramycin), gentamicin, tetracycline, ampicillin, piperacillin, trimethoprim, chloramphenicol, amoxicillin/ clavulanic acid, nitrofurantoin	*catA, nfsA, nfsB, par C, gyrA*	Lauteri et al.[290]

S. Typhimurium<br>*S. Enteritidis*<br>*S. Infantis*	Turkey	Poultry	tetracycline,<br>trimethoprim/Sulfamethoxazole,<br>ampicillin	*Tet(A),*<br>*sul1,*<br>*blaTEM*	Arkali et al.[291]
S. enteritidis	Iran	Cattle	gentamicin,<br>streptomycin,<br>sulfamethoxazole,<br>amikacin,<br>ceftriaxone,<br>imipenem	*bla*IMP-1, *tet(B)*	RanJbar et al.[292]
S. typhimurium	Czech Republic	Pigs	streptomycin, sulphonamides, ampicillin, tetracycline, chloramphenicol	*blaPSE-1, floR, aadA2, sul1, tet(G)*	Sisak et al. [293]
Staphylococcus aureus	Poland	Poultry, slaughterhouses	penicillin G, amoxicillin, cefoxitin, amoxicillin + clavulanic acid, tetracycline, erythromycin, clindamycin	*mecA*	Marek et al.[294]
	Japan	Chicken, beef, pork meat	ampicillin, erythromycin, clindamycin, levofloxacin	*blaZ, erm(C)*	Osada et al.[295]
	Turkey	Human	oxacillin<br>gentamicin<br><br>erythromycin<br><br>tetracyclin<br>penicillin	*mecA*<br>*aac(6')/aph(2''), aph(3'-IIIa, ant(4')-Ia*<br>*ermA, ermB, ermC, and msrA*<br>*tet(K), tet(M)*<br>*blaZ*	Duran et al. [296]
		Humans,	erythromycin,<br>ampicillin,	*mecA, erm(B), erm(C), vanB, vanA, vanC,*	Osei Sekyere & Mensah, [275]

	Southern, West and Northen Africa		penicillin, trimethoprim/sulfamethoxazole, tetracycline	*tet(O), tet(M), tet(K), tet(L),*	
		Animals	penicillin, clindamycin, ampicillin, trimethoprim/sulfamethoxazole, vancomycin, erythromycin, ciprofloxacin		
		Environment	penicillin, sulfamethoxazole/trimethoprim, ampicillin, vancomycin, erythromycin, ciprofloxacin, clindamycin		
	Ethiopia	Human,<br><br>vegetables	B-lactams, aminoglicosides, phenicols, lincosamides, macrolides, fluoroquinolones, tetracyclines, oxazolidiones, annamycin, sulfonamides, quinolones, glycopeptides, nitrofurantoins	*erm A, ermB, erm C, msrA*	Yigrem e2al.[297]
S.haemolyticus	India	Environment, foods	cefoxitin, erythromycin, co-trimoxazole, clindamycin, tetracyclines	*mecA*<br>*dfrG, ermC, msrAB, cfr, tet(K),*	Manoharan et al.[298]
Staphylococcus spp.	Iran	Ready-to-eat food chicken, beef, plant	penicillin, tetracycline, gentamicin, erythromycin, trimethoprim-sulfamethoxazole, ciprofloxacin	*Tet(K), blaZ, aacA-D, gyrA, ermA*	Mesbah et al.[299]
Staphylococcus spp.	Czech Republic	Dairy milk and beef meat	erythromycin, penicillin, tetracycline,	*erm(A), erm(B), erm(C), mph(C) msr(A), mph(C)*	Schlegelova et al.[300]

S. aureus, S. hyicus, S. epidermedius, S. lugdunensis, S. haemolyticus, S. hominus, S. schleiferi, S. cohnii, S. intermedius, and S. lentus	Egypt	Chicken meat	penicillin, gentamycin, clindamycin, oxacillin, and sulfamethoxazole/trimethoprim	*mecA, gyrA, gyrB, grlA*	Osman et al.[301]
S. epidermidis, S. intermedius, S. hyicus, S. aureus, S. caprae, S. gallinarum, three S. simulans, S. saprophyticus	Brazil	Goat, sheep-mastitis	amoxicillin, streptomycin, tetracycline, lincomycin, erythromycin, rifampicin, oxacillin, norfloxacin, doxycicline, ciprofloxacin, enrofloxacin, gentamycin	*blaZ, ermA, ermB, ermC, icaD*	Franca et al. [302]

trimethoprim (*dfrA17 and dfrA5*) and streptomycin/spectinomycin (*aadA5*); aminoglycosides (*aadA and strB*), β-lactams (*blaGES, blaOXA, blaVEB*), macrolide–lincosamide–streptogramin B (MLSB) (*ereA, ermF, and matA/mel*), sulfonamides (sul1), tetracyclines (*tet)*, and multidrug resistance (*qacEdelta1 and qacH*)
